# Proteome Analysis of the Plasma Membrane of *Mycobacterium Tuberculosis*

**DOI:** 10.1002/cfg.211

**Published:** 2002-12

**Authors:** Sudhir Sinha, Shalini Arora, K. Kosalai, Abdelkader Namane, Alex S. Pym, Stewart T. Cole

**Affiliations:** 1 Division of Biochemistry Central Drug Research Institute Chattar Manzil Post Box No. 173 Lucknow 226001 India; 2 PT3 — Protéomique Génopole Institut Pasteur 28 Rue du Docteur Roux Paris Cedex 15 75724 France; 3 Unité de Génétique Moléculaire Bactérienne Institut Pasteur 28 Rue du Docteur Roux Paris Cedex 15 75724 France

## Abstract

The plasma *membrane of Mycobacterium tuberculosis* is likely to contain proteins that could serve as novel drug targets, diagnostic probes or even components of a vaccine against tuberculosis. With this in mind, we have undertaken proteome analysis of the membrane of *M. tuberculosis* H37Rv. Isolated membrane vesicles were extracted with either a detergent (Triton X114) or an alkaline buffer (carbonate) following two of the protocols recommended for membrane protein enrichment. Proteins were resolved by 2D-GE using immobilized pH gradient (IPG) strips, and identified by peptide mass mapping utilizing the *M. tuberculosis* genome database. The two extraction procedures yielded patterns with minimal overlap. Only two proteins, both HSPs, showed a common presence. MALDI–MS analysis of 61 spots led to the identification of 32 proteins, 17 of which were new to the *M. tuberculosis* proteome database. We classified 19 of the identified proteins as ‘membrane-associated’; 14 of these were further classified as ‘membrane-bound’, three of which were lipoproteins. The remaining proteins included four heat-shock proteins and several enzymes involved in energy or lipid metabolism. Extraction with Triton X114 was found to be more effective than carbonate for detecting ‘putative’ *M. tuberculosis* membrane proteins. The protocol was also found to be suitable for comparing BCG and *M. tuberculosis* membranes, identifying ESAT-6 as being expressed selectively in *M. tuberculosis*. While this study demonstrates for the first time some of the membrane proteins of *M. tuberculosis*, it also underscores the problems associated with proteomic analysis of a complex membrane such as that of a mycobacterium.


**Comparative and Functional Genomics**
*Comp Funct Genom* 2002; **3**: 470–483.
Published online 25 November 2002 in Wiley InterScience (www.interscience.wiley.com). **DOI:** 10.1002/cfg.211

**Research Article**

### Proteome analysis of the plasma membrane of *Mycobacterium tuberculosis*

Sudhir Sinha[1]*, Shalini Arora[1], K. Kosalai[1], Abdelkader Namane[2], Alex S. Pym[3] and Stewart T. Cole[3]

[1] *Division of Biochemistry, Central Drug Research Institute, Chattar Manzil, Post Box No. 173, Lucknow 226001, India*
[2] *PT3 — Protéomique Génopole, Institut Pasteur, 28 Rue du Docteur Roux, 75724 Paris Cedex 15, France*
[3] *Unité de Génétique Moléculaire Bactérienne, Institut Pasteur, 28 Rue du Docteur Roux, 75724 Paris Cedex 15, France*

**Correspondence to:*
*Sudhir Sinha, Division of Biochemistry, Central Drug Research Institute, PO Box 173, Lucknow 226001, India.*
*E-mail: sinhas@lycos.com*

### Abstract

**The plasma membrane of *Mycobacterium tuberculosis* is likely to contain proteins that could serve as novel drug targets, diagnostic probes or even components of a vaccine against tuberculosis. With this in mind, we have undertaken proteome analysis of the membrane of *M. tuberculosis* H37Rv. Isolated membrane vesicles were extracted with either a detergent (Triton X114) or an alkaline buffer (carbonate) following two of the protocols recommended for membrane protein enrichment. Proteins were resolved by 2D-GE using immobilized pH gradient (IPG) strips, and identified by peptide mass mapping utilizing the *M. tuberculosis* genome database. The two extraction procedures yielded patterns with minimal overlap. Only two proteins, both HSPs, showed a common presence. MALDI–MS analysis of 61 spots led to the identification of 32 proteins, 17 of which were new to the *M. tuberculosis* proteome database. We classified 19 of the identified proteins as 'membrane-associated'; 14 of these were further classified as 'membrane-bound', three of which were lipoproteins. The remaining proteins included four heat-shock proteins and several enzymes involved in energy or lipid metabolism. Extraction with Triton X114 was found to be more effective than carbonate for detecting 'putative' *M. tuberculosis* membrane proteins. The protocol was also found to be suitable for comparing BCG and *M. tuberculosis* membranes, identifying ESAT-6 as being expressed selectively in *M. tuberculosis*. While this study demonstrates for the first time some of the membrane proteins of *M. tuberculosis*, it also underscores the problems associated with proteomic analysis of a complex membrane such as that of a mycobacterium. Copyright © 2002 John Wiley & Sons, Ltd.**

Received: 6 June 2002
Accepted: 11 September 2002

**Keywords:** genome; proteome; *Mycobacterium tuberculosis*; membrane; peptide mass mapping

### Introduction

A third of the world's population is considered to be infected with *Mycobacterium tuberculosis*, which leads to nearly 8 million new patients and 3 million deaths due to tuberculosis every year. Multi-drug-resistant strains of the pathogen, emerging in association with HIV, have added a frightening dimension to the problem (Raviglione *et al*., 1995; Snider and Castro, 1998). It has long been realized that successful tuberculosis control strategies would evolve from exploring and exploiting the biology of *M. tuberculosis* (Young and Duncan, 1995; Cole *et al*., 1998). Identification and characterization of subcellular proteins of the microbe is important to understanding their function, especially where the genomic data is not predictive or, quite often, not even suggestive (Domenech *et al*., 2001; Jungblut *et al*., 2001). Covalent modifications to amino acid sequences can occur co- or post-translationally, and neither event is dictated, at least not in a manner that is fully understood, by the nucleotide sequences of genes (Jensen, 2000). Other types of processing events, such as

Copyright © 2002 John Wiley & Sons, Ltd.

proteolytic cleavage, may need to occur before a protein is rendered fully functional. Furthermore, proteins can function as multimeric complexes, hence identifying the interacting partners involved in these complexes is essential to understanding biological processes. Consequently, a systematic and comprehensive study of the proteins of the pathogen is required in the quest for novel drug targets, diagnostic probes or vaccines (Monahan *et al.*, 2001; Covert *et al.*, 2001; Betts *et al.*, 2000; Rosenkrands *et al.*, 2000a,b). Proteome analysis, involving a combination of 2D electrophoresis, mass spectrometry, and bioinformatics, has now emerged as a robust and efficient strategy for rapid identification of proteins (Jungblut *et al.*, 1999).

The prokaryotic cell membrane is rich in proteins comprising several essential enzymes, receptors and transporters (Brennan and Nikaido, 1995; Sigler and Hofer, 1997). Being lipid in nature, it also provides a hydrophobic environment for certain biochemical reactions. Immunogenicity of membrane proteins, as reported with certain pathogens, has been attributed to their inherent hydrophobicity and lipid modification (Deres *et al.*, 1989; Akins *et al.*, 1993; Frankenburg *et al.*, 1996). Bioinformatic analysis of the *M. tuberculosis* genome predicts >65 lipoproteins of 'cell envelope' origin, some of which were identified previously as 'secreted' proteins, or enzymes involved in cell wall biogenesis. Besides these, there are 17 conserved MmpL and MmpS proteins, and >600 other 'putative' membrane proteins. The latter differ in the number of transmembrane hydrophobic segments, and include proteins belonging to the major facilitator and ATP-binding cassette (ABC) superfamilies (Tekaia *et al.*, 1999). These proteins undoubtedly play a role in the uptake and effects of various metabolites, peptides, drugs and antibiotics. Nonetheless, the real location, expression patterns and function of most of these proteins remain unexplored (Young and Garbe, 1991; Lee *et al.*, 1992).

The aim of our study is to characterize mycobacterial membrane proteins. Upon extraction with Triton X114 (Bordier, 1981), they partition into detergent- and water-rich phases, the former being classified as 'integral' and the latter as 'peripheral' membrane proteins (IMP and PMP; Mehrotra *et al.*, 1995). In both BCG (Mehrotra *et al.*, 1999) and *M. tuberculosis* H37Rv (unpublished) the IMP pool was found to contain, besides a range of unidentified proteins, three of the known 'immunodominant' ones: the 19 and 38 kDa lipoproteins and the 33/36 kDa 'proline-rich' protein. Similarly, both the PMP pools showed the presence of the 16 kDa $\alpha$-crystallin heat-shock protein. Of particular interest are the results of human T cell proliferation assays, which showed a significantly higher T cell activating potency of IMP over PMP or cytosol. These results focused our attention on IMP and we applied a protocol based on 'continuous-elution SDS–PAGE' for purification of its constituents (Mehrotra *et al.*, 1997). Purified fractions were compared in human T cell proliferation assays, which revealed a greater T cell stimulatory potency of certain low molecular weight IMPs than the others (Mehrotra *et al.*, 1999).

There is thus enough evidence to suggest that identification of *M. tuberculosis* membrane proteins, and monitoring changes in their expression profiles under relevant conditions of growth, would be of considerable importance for designing novel and effective interventions against tuberculosis. However, despite some recent progress in this direction (Molloy *et al.*, 2000), proteome analysis of the cell membrane remains a challenge, due mainly to problems encountered during the resolution of membrane-associated proteins by the available 2D electrophoresis protocols (Santoni *et al.*, 2000). In this paper we describe the results of our proteomic analysis of the membrane of *M. tuberculosis* H37Rv using 2D electrophoresis and peptide mass mapping. Proteins for this purpose were obtained by extraction of membrane vesicles with either Triton X114 or carbonate (Fujiki *et al.*, 1982). We have also tried to ascertain the suitability of the protocols used for proteomic comparisons between mycobacterial membranes.

### Materials and methods

### Isolation and fractionation of mycobacterial cell membranes

The membranes of *M. tuberculosis* H37Rv and *M. bovis* BCG (Pasteur) were isolated as described previously (Mehrotra *et al.*, 1995; Mehrotra *et al.*, 1999). In brief, 3–4 week old bacterial cultures (in Lowenstein–Jensen medium) were harvested and probe-sonicated in a buffer containing protease inhibitors (50 mM Tris, 10 mM $MgCl_2$, 1 mM EGTA, 1 mM PMSF, 0.02% $NaN_3$, pH 7.4). The sonicates were centrifuged, initially at $23\,000 \times g$

Copyright © 2002 John Wiley & Sons, Ltd. 

to remove cell wall debris and later at 150 000 × $g$ to obtain the membrane sediment. The sediment was resuspended in sonication buffer and membrane vesicles were morphologically characterized by transmission electron microscopy (Mehrotra *et al.*, 1995). Proteins from washed vesicles were extracted using either of the following protocols. In the first protocol, the membrane sediment was resuspended in sonication buffer at a concentration of 10 mg protein/ml, to which precondensed Triton X114 (Bordier, 1981) was added, at a final concentration of 2%. The suspension was then stirred (1 h, 4 °C) to obtain the extract in a single phase. Residual insoluble matter was removed by centrifugation at 150 000 × $g$ and the phases were allowed to separate at 37 °C in a water bath. The upper (aqueous) and lower (detergent) phases were collected by centrifugation (1000 × $g$) and 'back-washed' three times. Protein in the pooled detergent phase was recovered by precipitation with 5 volumes of chilled acetone. Dried sediment was resuspended in distilled water and stored as lyophilized aliquots. In the second protocol, membrane components were extracted with carbonate using the recommended procedure (Fujiki *et al.*, 1982). In brief, the membrane pellet was suspended in 100 mM sodium carbonate (pH 11.5) to give a final protein concentration of 1 mg/ml. It was stirred for 30 min at 4 °C and centrifuged at 150 000 × $g$ for 1 h at 4 °C. Sediment (membrane 'stripped' of the peripheral proteins) was resuspended in distilled water and stored in lyophilized aliquots. All protein estimations were done by the modified Lowry method (Markwell *et al.*, 1978).

### Two-dimensional polyacrylamide gel electrophoresis (2D-PAGE)

A solubilization buffer (7 M urea, 2 M thiourea, 4% CHAPS, 0.3% DTT, 2% carrier ampholytes 3–10 and 40 mM Tris, pH 9.6) recommended for isoelectric focusing (IEF) of membrane proteins (Friso and Wikstrom, 1999) was used. 1 mg lyophilized protein was solubilized in 500 µl this buffer by vortexing (30 min). This was followed by centrifugation (12 000 rpm × 10 min) to remove the insoluble matter. 150 µl (∼300 µg protein) or 450 µl (∼900 µg protein) clear supernatant was applied, respectively, to immobilized pH gradient (IPG) strips of 7 cm or 17 cm length (Bio-Rad), using the method of 'in-gel rehydration' (Gorg *et al.*, 2000). IEF was performed at 20 °C in an IEF cell (Bio-Rad) using the following four-step program for 7 cm IPG strips: (a) 0–250 V in 1 h; (b) 250 V constant for 1 h; (c) 250–3000 V in 4 h; and (d) 3000 V constant until 15 kVh. In the case of the 17 cm strips, the applied voltage gradient was as follows: (a) 0–250 V in 1 h; (b) 250 V constant for 2 h; (c) 250–3000 V in 5 h; (d) 3000 V constant until 50 kVh. The current limit was set at 50 µA/strip. In each case, the program led to a 'steady state'.

After IEF, IPG strips were equilibrated sequentially (Gorg *et al.*, 2000) in solutions 'A' (0.05 M Tris–HCl, pH 8.8, containing 6 M urea, 30% glycerol, 2% SDS, 1% DTT) and 'B' (0.05 M Tris–HCl, pH 8.8, containing 6 M urea, 30% glycerol, 2% SDS, 4% iodoacetamide, 0.005% bromophenol blue). Later, each strip was loaded on top of a vertical SDS–polyacrylamide gel (12% T, 1.08% C, 1 mm thickness) and sealed in place with 1% low-melting agarose dissolved in electrode buffer. Molecular mass markers were loaded in a separate well by the side of the strip. Electrophoresis was performed using Laemmli's (1970) buffer system at a constant current of 20 mA (for 7 cm gels) or 40 mA (for 17 cm gels) until the indicator dye (bromophenol blue) approached the bottom edge of the gels. Proteins were stained with Coomassie brilliant blue R250. Images were acquired by an imaging densitometer (GS710, Bio-Rad).

### Peptide mass mapping

For in-gel digestion, sample preparation was performed as described by Shevchenko *et al.* (1996). Briefly, the Coomassie blue stained spot was excised from the gel, washed, in-gel reduced, S-alkylated with iodoacetamide and digested with bovine trypsin (sequencing grade, Roche Molecular Biochemicals) at 37 °C overnight. Peptides were extracted, dried in a SpeedVac and resolubilized in 8 µl 0.1% TFA. ZipTips (Millipore) were used to desalt the samples. Peptide mass mapping was performed on 0.5 µl tryptic digest mixture using $\alpha$-cyano-4-hydroxycinnamic acid (CHCA, Sigma). The samples were analysed by MALDI–MS on a Voyager DE STR (PerSeptive Biosystems, Framingham, MA, USA) equipped with a nitrogen laser (337 nm). The instrument was operated in the delayed extraction mode with a delay time of 150 ns. Each mass spectrum was

Copyright © 2002 John Wiley & Sons, Ltd. 

an average of 250 laser shots. Close external calibration was performed with a mixture of des-arg-1 bradykinin (904.468), angiotensin-I (1296.685), neurotensin (1672.917), and fragment 18–39 of adrenocorticotropic hormone (2465.199). To search the *Mycobacterium tuberculosis* ORF database 'TubercuList' (**http://genolist.pasteur.fr/TubercuList**), monoisotopic masses were assigned, using a local copy of the MS-Fit3.2 part of the Protein Prospector package (University of California, Mass Spectrometry Facility, San Francisco, CA). The parameters were set as follows: no restriction on the isoelectric point of proteins, a maximum mass error of 100 ppm and only one incomplete cleavage allowed per peptide.

### Results

### 2D resolution patterns *of M. tuberculosis* membrane proteins

Despite using a widely accepted protocol for membrane solubilization, a portion of the sample remained insoluble and formed sediment upon centrifugation. With this limitation, the visibility of spots after Coomassie blue staining was best at the applied protein concentrations. Lesser amounts produced poorly visible spots and greater amounts led to an increase in streaking and smearing. Silver staining might have detected more spots, but was not used since it could have posed problems during protein identification by MALDI–MS. The reproducibility of the 2D patterns was confirmed and considered final when two consecutive runs produced identical patterns.

2D resolution patterns of the detergent (Triton X114) soluble membrane proteins of *M. tuberculosis* are shown in Figures 1 and 2. With the use of a pH 3–10 IPG strip (Figure 1), proteins in the mass range of 35–80 kDa tended to focus sharply in the neutral-to-basic region of the gel. However, separation was not so clear in the acidic region, particularly of proteins of 15–35 kDa. Similar results were obtained with a pH 4–7 IPG

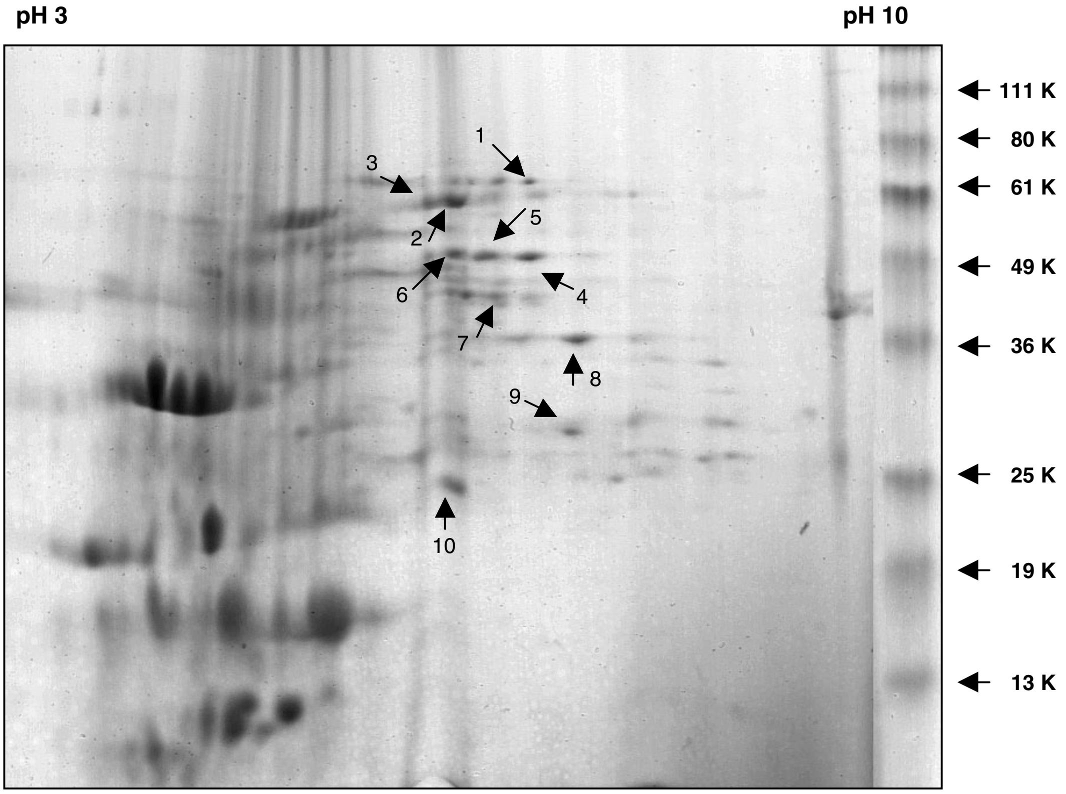

**Figure 1.** 2D gel electrophoresis of Triton X114-soluble membrane proteins of *M. tuberculosis* H37Rv. Proteins were separated by isoelectric focusing on pH 3–10 IPG strips and then by SDS–PAGE on 12.5% Laemmli gels. Staining was done with Coomassie blue. Spots 1–10 were processed for protein identification by peptide mass mapping

Copyright © 2002 John Wiley & Sons, Ltd.

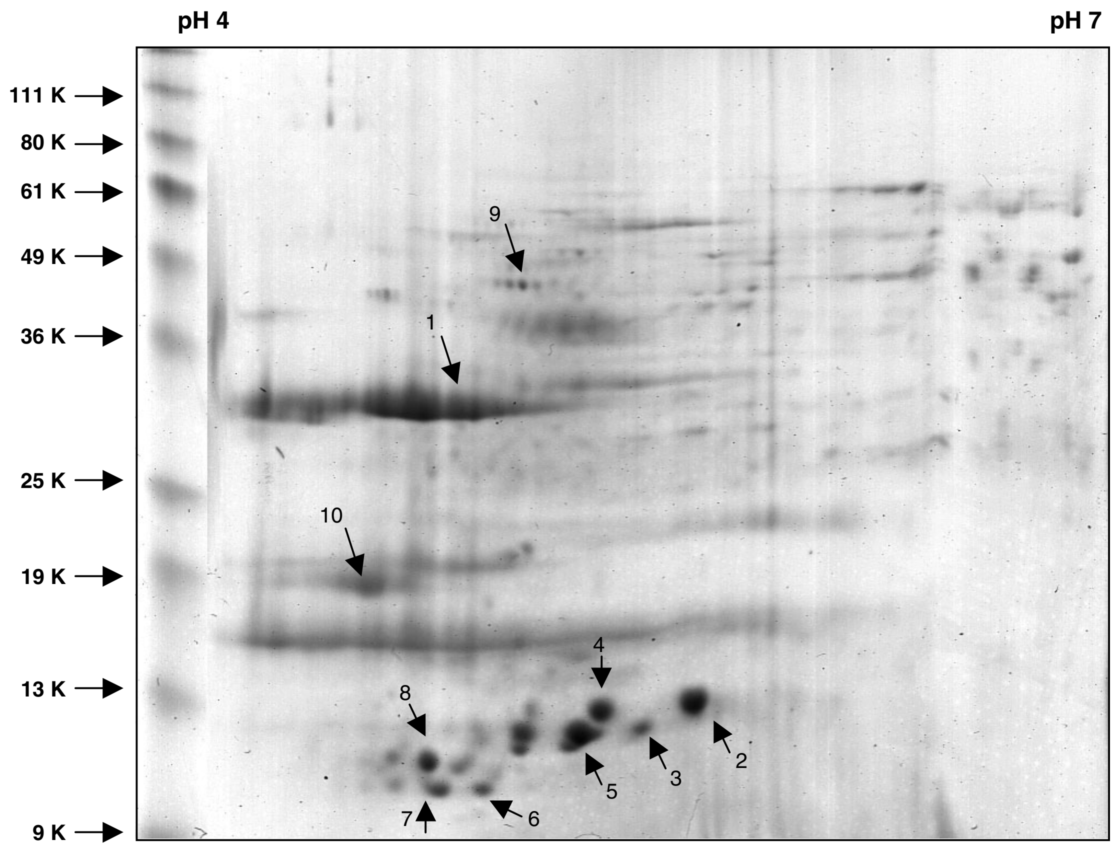

**Figure 2.** 2D-GE of Triton X114 soluble membrane proteins of *M. tuberculosis* H37Rv. Proteins were separated using pH 4–7 IPG strips and SDS–PAGE on 12.5% gels. Spots 1–10 were processed for protein identification strip (Figure 2), wherein relatively larger proteins (35–80 kDa) focused mostly towards neutral pH and smaller ones (1535 kDa) appeared mostly unresolved. In addition, this gel also showed good resolution of certain high abundance, low molecular mass (<15 kDa) proteins in the acidic region.

2D profiles of the carbonate-extracted membrane sample (Figures 3 and 4) appeared different from those of the detergent soluble sample. With either pH 3–10 (Figure 3) or pH 4–7 (Figure 4) IPG strips, proteins of 25–40 kDa appeared grossly unresolved in the acidic region of gel, though the smaller (<15 kDa) proteins focused sharply. Several proteins of a relatively higher molecular mass (>20 kDa) also tended to focus sharply in the basic region of gel.

### Protein identification by peptide mass mapping

Sixty-one protein spots (marked in Figures 1–4) isolated from *M. tuberculosis* membranes were picked for identification; 20 of these were from the detergent-soluble samples (Figures 1 and 2) and 41 were from the carbonate extracted samples (Figures 3 and 4). Selection was generally based on clarity and the ease with which the spots could be picked. For individual spots, the number of peptides giving matches in the genome database were in the range 3–18 and the coverage 8–60%. Although 8% sequence coverage may be considered low, all five peptides of this particular protein (Rv0822c, Table 1) come from the N-terminal domain. Its molecular mass as estimated from the 2D gel analysis was about 15 kDa (Figure 2, spot 4) whereas the predicted mass is 72 911 Da. This probably indicates proteolysis of this protein, causing liberation of an N-terminal domain that was well covered by the peptides detected by mass spectrometry.

Peptide mass mapping of the selected spots led to the identification of 32 proteins, 15 of which

Copyright © 2002 John Wiley & Sons, Ltd. 

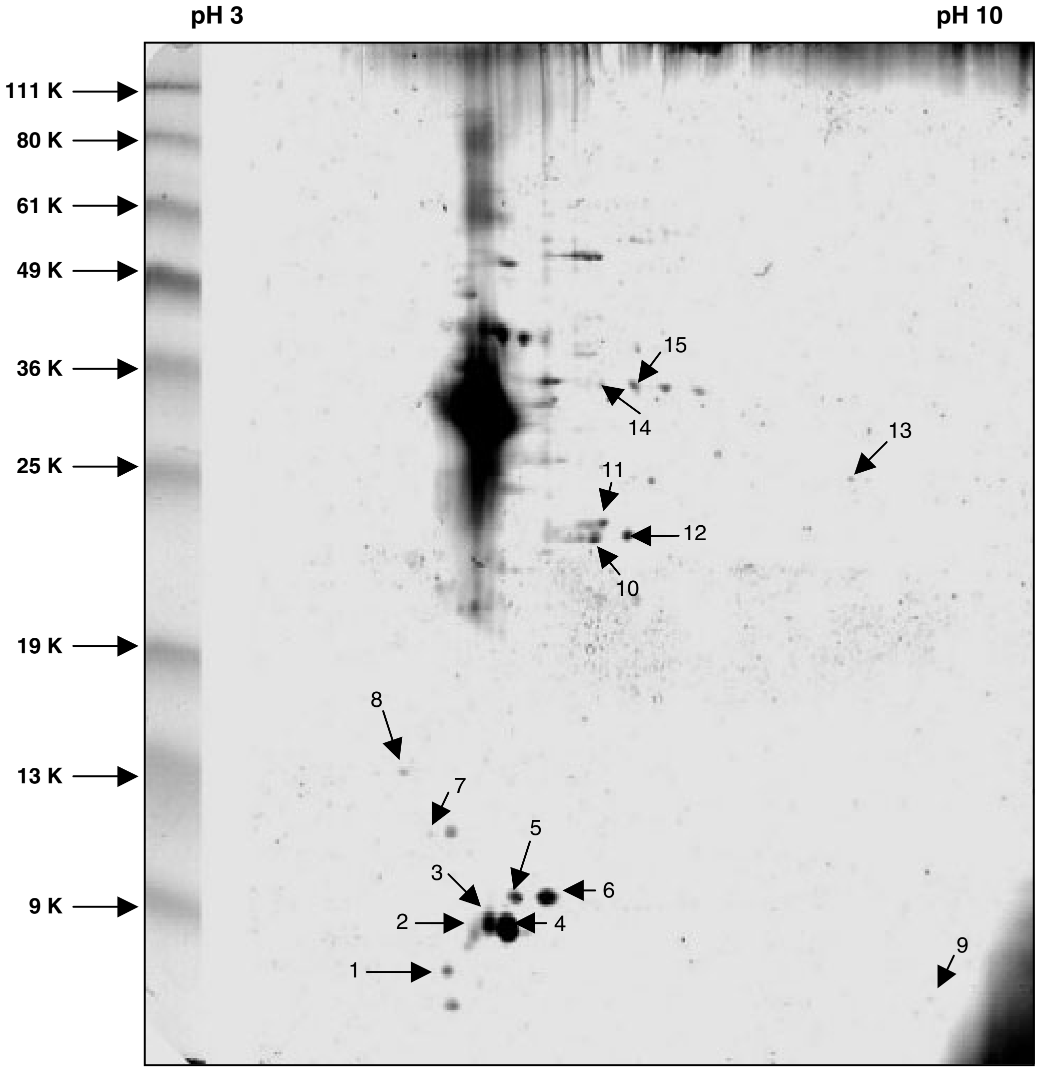

**Figure 3.** 2D-GE of carbonate-extracted membrane of *M. tuberculosis* H37Rv. Proteins were separated by using pH 3–10 IPG strips and SDS–PAGE on 12.5% gels. Spots 1–15 were processed for protein identification originated from the detergent soluble samples and 19 from carbonate extracted membrane samples (Tables 1–3). Only two proteins were common to both preparations (Rv2031c, Table 2 and Rv0251c, Table 3). In all, 17 proteins (eight in Table 1, three in Table 2 and six in Table 3) were considered 'new' to the *M. tuberculosis* proteome database, since they had not been reported previously.

The identified proteins were classified into three categories: (a) putative membrane proteins ($n = 14$, Table 1); (b) proteins with known membrane associations ($n = 5$, Table 2); and (c) other proteins ($n = 13$, Table 3). The criteria used for this classification were published reports, annotations in the genome database (**http://genolist.pasteur.fr/TubercuList**), and predictions for transmembrane regions, signal sequences or lipid attachment sites by two relevant prediction servers TMPRED (**http://www.ch.embnet.org/software/TMPRED_form.html**) and PSORT (**http://psort.nibb.ac.jp**). Nineteen proteins (60% of all those identified) met the criteria to be described as 'membrane-associated' (Tables 1 and 2). 'Grand average of hydropathy' (GRAVY) scores for all proteins were obtained using ProtParam (**http://us.expasy.org/tools/protparam.html**), in which a score $>-0.4(=$

Copyright © 2002 John Wiley & Sons, Ltd. 

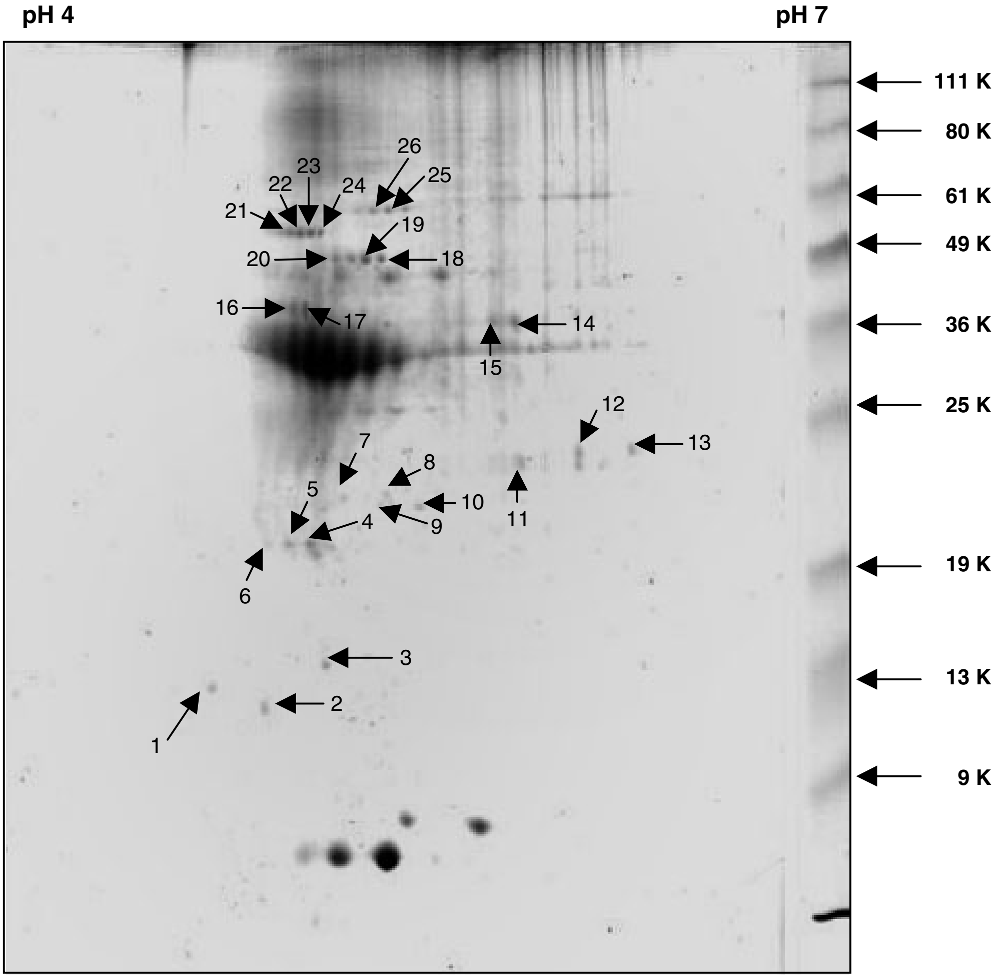

**Figure 4.** 2D-GE of carbonate-extracted membrane of *M. tuberculosis* H37Rv. Proteins were separated using pH 4–7 IPG strips and SDS–PAGE on 12.5% gels. Spots 1–26 were processed for protein identification mean score for cytosolic proteins) indicates probability for membrane association — the higher the score greater the probability (Kyte and Doolittle, 1982). By this criterion alone, 25 (78%) proteins could be considered 'hydrophobic' since only seven (two in Table 2, and five in Table 3) showed a GRAVY score of $< -0.4$.

Detergent solubilization appeared to perform better than carbonate extraction in isolating membrane proteins. By the most stringent criteria applied in this study (Table 1), 9/15 (60%) proteins identified from the detergent-soluble samples and only 5/19 (26%) proteins from the carbonate-extracted membrane qualified as 'membrane-bound'. Further, the analysis of detergent-soluble, but not carbonate-extracted, membrane samples also revealed three lipoproteins of the bacillus (Rv1270c, Rv1368 and Rv0934).

The observed mass and charge of several proteins were different from those predicted by the genome, which is a common feature of most proteomic analyses, probably reflecting the effect of protein 'maturation' events including co- or post-translational modifications. Likewise, many proteins appeared as more than one spot representing their multiple 'charge' and/or 'mass' forms. In this respect, a significant observation was the presence of multiple forms of the FadA2 protein (Rv0243). The database predicts the mass of this protein as 46 075 Da and the pI as 6.64. However the expressed mature protein appeared to have three charge forms (Figure 1, spots 4–6) and one mass form (Figure 1, spot 9),

Copyright © 2002 John Wiley & Sons, Ltd. 

**Table 1.** Putative membrane proteins

| Fig. No.; spot No. | Protein | Peptides matched/ sequence covered | Size (Da), pI | Functional category+/description | Predicted TM/LP/SS* | GRAVY score@ | Previous proteomic detections# |
|---|---|---|---|---|---|---|---|
| Fig. 1; 1 | Rv0270 | 16/36% | 59 909, 6.76 | 1/Probable FadD2 | TM = 272–290$^{a}$ | −0.071 | [i] |
| Fig. 1; 3 | Rv2682c | 9/21% | 67 886, 6.34 | 7/Dxs, probable 1-deoxyxylulose 5-phosphate synthase | TM = 367–387$^{a}$, 503–519$^{a,b}$ | 0.024 | [ii] |
| Fig. 1; 4–6, 9 | Rv0243 | 7–15/21–40% | 46 075, 6.64 | 1/FadA2 | TM = 422–438$^{a,b}$ | −0.050 | — |
| Fig. 1; 7 | Rv1770 | 10/25% | 45 964, 6.52 | 10/Unknown | TM = 95–111$^{a,b}$, 114–130$^{b}$, 228–244$^{a,b}$ | −0.025 | — |
| Fig. 1; 8 | Rv1872c | 18/34% | 45 333, 10.15 | 7/IldD2, probable oxidoreductase | TM = 126–142$^{a}$, 329–345$^{a,b}$ | 0.058 | — |
| Fig. 1; 10 | Rv1368 | 7/40% | 26 848, 9.15 | 3/Hypothetical. N-terminal signal sequence and lipid attachment site. Homologous to Rv1270c | TM = 20–36$^{a,b}$ | −0.032 | — |
| Fig. 2; 1 | Rv0934 | 4/18% | 38 207, 5.02 | 3/Mycobacterial equivalent of PstS, prokaryotic membrane lipoprotein, lipid attachment site at N-terminus | LP$^{b}$, TM = 7–23$^{a}$ | 0.066 | [ii, iii] |
| Fig. 2; 4 | Rv0822c | 5/8% | 72 911, 6.08 | 10/Similar to membrane bound regulatory protein from *B. subtilis* | TM = 169–192$^{a,b}$ | −0.278 | — |
| Fig. 2; 10 | Rv1270c | 6/27% | 24 871, 5.21 | 3/Similar to *M. bovis* 27 kDa lipoprotein | LP$^{b}$, TM = 8–25$^{a}$ | 0.014 | — |
| Fig. 3; 9 | Rv2159c | 12/37% | 36 363, 7.82 | 8/Unknown | TM = 306–329$^{a}$, 145–161$^{b}$ | −0.050 | — |
| Fig. 3; 14–15 Fig. 4; 2–3 | Rv2461c | 3–5/17–37% | 21 664, 4.54 | 7/ClpP, ATP-dependent clp protease proteolytic subunit | TM = 71–95$^{a}$, 81–108$^{a}$ | 0.039 | [iv] |
| Fig. 4; 10 | Rv3099c | 5/21% | 30 465, 5.02 | 8/Unknown | SS$^{b}$ | −0.073 | — |
| Fig. 4; 18–20 | Rv3417c | 5–7/17–23% | 55 894, 4.74 | 0/groEL-1 | TM = 103–119$^{a,b}$, 445–466$^{a}$ | 0.110 | [i] |
| Fig. 4; 21–24 | Rv0440 | 4–5/13–17% | 56 755, 4.56 | 0/groEL-2 | TM = 500–519$^{a}$ | −0.091 | [ii, iii] |

+ Functional categories: 0, virulence, detoxification, adaptation; 1, lipid metabolism; 2, information pathways; 3, cell wall and cell processes; 7, intermediary metabolism and respiration; 8, unknown; 9, regulatory proteins; 10, conserved hypotheticals.
* TM, transmembrane/hydrophobic regions; LP, lipoprotein; SS, signal sequence.
[a] Using the software TMPRED (**http://www.ch.embnet.org/software/TMPRED_form.html**).
[b] Using the software PSORT (**http://psort.nibb.ac.jp**).
@ According to Kyte and Doolittle (1982).
\# [i] Jungblut *et al.* (1999); [ii] Rosenkrands *et al.* (2000a); [iii] Rosenkrands *et al.* (2000b); [iv] Covert *et al.* (2001).

Copyright © 2002 John Wiley & Sons, Ltd.

**Table 2.** Proteins with known membrane associations

| Fig. No.; spot No. | Protein | Peptides matched/ sequence covered | Size (Da), pI | Functional category+/ description | Evidence for membrane association* | GRAVY score@ | Previous proteomic detections# |
|---|---|---|---|---|---|---|---|
| Fig. 2; 5<br>Fig 3; 1 | Rv2031c | 4–5/31–37% | 16 218, 4.75 | 0/HSP16, $\alpha$-crystallin homologue | [a,b,c] | −0.520 | [ii, iii] |
| Fig. 3; 13<br>Fig. 4; 1 | Rv1876 | 3–5/30–43% | 18 329, 4.23 | 7/BfrA, bacterioferritin. Also similar to bfrB, Rv3841 | [d] | −0.455 | — |
| Fig. 4; 11 | Rv1309 | 4/15% | 33 883, 5.15 | 7/AtpG, ATP synthase $\gamma$ chain | [e] | −0.267 | — |
| Fig. 4; 16–17 | Rv1310 | 14–16/43–46% | 53 097, 4.60 | 7/AtpD, ATP synthase $\beta$ chain | [e] | −0.168 | — |
| Fig. 4; 25–26 | Rv1308 | 5–9/9–21% | 59 305, 4.78 | 7/AtpA, ATP synthase $\alpha$ chain | [e] | −0.207 | [ii] |

+ As in Table 1.
* [a] Young and Garbe (1991); [b] Lee *et al.* (1992); [c] Mehrotra *et al.* (1999); [d] Pessolani *et al.* (1994); [e] Futai *et al.* (1989).
@ As in Table 1. Scores <−0.4, suggesting greater probability for cytosolic origin, are underlined.
### As in Table 1.

**Table 3.** Other proteins

| Fig. No.; spot No. | Protein | Peptides matched/ sequence covered | Size (Da), pI | Functional category+/ description | GRAVY score@ | Previous proteomic detections# |
|---|---|---|---|---|---|---|
| Fig. 2; 2,4<br>Fig. 3; 2–3 | Rv0251c | 5–8/48–59% | 17 774, 5.04 | 0/Probably HSP20 family | −0.473 | — |
| Fig. 2; 3 | Rv3547 | 4/42% | 17 364, 9.94 | 10 | −0.544 | — |
| Fig. 2; 7 | Rv0784 | 6/37% | 25 006, 10.15 | 10 | 0.075 | — |
| Fig. 2; 8 | Rv0689c | 3/60% | 8735, 4.99 | 8 | −0.458 | — |
| Fig. 2; 9 | Rv1023 | 11/35% | 44 925, 4.26 | 7/Probable enolase, similar to ENO_ECOLI | 0.015 | — |
| Fig. 3; 4–5 | Rv2744c | 4–5/22–31% | 29 255, 5.77 | 10/35 kDa antigen. CDS is split into two ORFs in *M. leprae* | −0.464 | [ii] |
| Fig. 3; 6.<br>Fig. 4; 12–13 | Rv2005c | 4/15% | 30 977, 5.62 | 10 | −0.458 | [i] |
| Fig. 3; 7 | Rv2296 | 6/26% | 33 347, 6.98 | 7/Hydrolase, similar to e.g. HALO_XANAU, haloalkane dehalogenase | −0.228 | [ii] |
| Fig. 3; 8 | Rv1479 | 8/35% | 40 755, 6.36 | 9/MoxR, highly similar to MoxR homologues of *M. tuberculosis*, Rv3692, Rv3164c and *M. avium* | 0.051 | [ii] |
| Fig. 3; 10–12 | Rv0242c | 6–10/19–30% | 46 797, 6.38 | 1/FabG4, probably 3-O xyloacyl-[acyl carrier protein] reductase | 0.069 | [ii] |
| Fig. 4; 4–6 | Rv0798c | 4–6/25–38% | 28 822, 4.67 | 0/29 kDa antigen, Cfp29 | −0.141 | [ii, iii] |
| Fig. 4; 7–9 | Rv0831c | 3–5/21% | 30 177, 4.85 | 8 | −0.212 | — |
| Fig. 4; 14–15 | Rv0685 | 6–12/26–46% | 43 567, 5.12 | 2/Tuf elongation factor Tu (ef-tu), ATP/GTP-binding motif A | −0.288 | [ii, iii] |

+ As in Table 1.
@ As in Table 1. Scores <−0.4, suggesting greater probability for cytosolic origin, are underlined.
### As in Table 1.

the latter being significantly smaller than the predicted size (Table 1).

Four heat-shock proteins were also found to be present in the membrane fractions. Among those present in both detergent-soluble and carbonate-extracted samples were an $\alpha$-crystallin homologue (Rv2031c, Table 2) and HSP20 (Rv0251c, Table 3). Two proteins of the HSP60 family,

Copyright © 2002 John Wiley & Sons, Ltd. 

groEL-1 and -2 (Rv3417c and Rv0440, Table 1), were seen only in the carbonate-extracted sample.

### Proteomic comparison of *M. tuberculosis* and BCG membranes

We were interested to know whether the proteome analysis protocol applied in this study was applicable to the identification of those proteins that are differentially expressed between mycobacterial cell membranes. The 2D protein profiles of the whole unfractionated membranes of *M. tuberculosis* and BCG that were obtained appeared quite similar (Figure 5). Peptide mass mapping of a low molecular mass protein (marked in Figure 5), which was expressed abundantly and selectively in the *M. tuberculosis* membrane, identified it as ESAT-6, which belongs to the family of 'early secretory antigenic target' proteins of the bacillus.

### Discussion

Proteome analysis of the cell membrane is considered a daunting task, due mainly to problems encountered at two levels: (a) isolation of membrane which is free from 'non-membrane' constituents; and (b) solubilization of membrane proteins in a manner amenable to isoelectric focusing (Santoni *et al*., 2000). The existing databases of the mycobacterial proteome (**http://www.ssi.dk/publichealth/tbimmun; http://www.mpiib-berlin.mpg.de/2D-PAGE/**) and some recent reports (Monahan *et al*., 2001; Covert *et al*., 2001; Mattow *et al*., 2001a,b) have so far identified nearly 500 proteins, yet hardly any of them correspond to the ~ 800 putative membrane proteins predicted from the genome. Against this backdrop, our work was planned as a case study on the application of proteomics to the membrane of *M. tuberculosis* H37Rv. The difference between our strategy and that of the previous studies is that we worked with isolated membrane fractions of the bacillus rather than the whole cell lysate. This fraction was further enriched for membrane proteins using two of the recommended protocols, involving extractions with either a detergent (Triton X114; Bordier, 1981) or carbonate (Fujiki *et al*., 1982). We also used a sample solubilization protocol specifically recommended for membrane proteins (Friso and Wikstrom, 1999). Another membrane solubilization protocol involving the use of sulphobetaine 3–10 and tributylphosphine (Molloy *et al*., 1998) was also tried, which gave similar results (not described here).

2D gel electrophoresis and peptide mass mapping of 61 spots from the two membrane samples of *M. tuberculosis* led to identification of

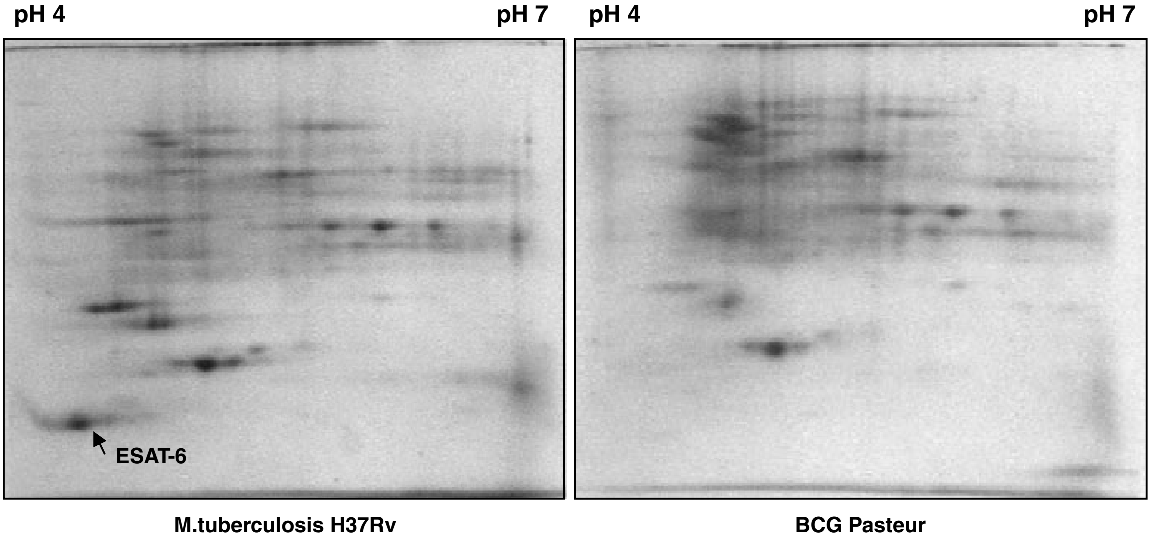

**Figure 5.** 2D-GE of whole cell membranes of *M. tuberculosis* H37Rv and BCG Pasteur. Isoelectric focusing was performed on pH 4–7 IPG strips and SDS–PAGE on 12.5% Laemmli gels. The marked spot expressed selectively in *M. tuberculosis* was processed for peptide mass mapping to be identified as ESAT-6

Copyright © 2002 John Wiley & Sons, Ltd.

32 proteins; 17 of these are 'new' to the mycobacterial protein database, as they bear no previous references. A set of parameters including published reports, annotations in the genome data, and software-based predictions for putative transmembrane/hydrophobic domains, signal sequences and lipid attachment sites were used for ascertaining the membranous nature of the identified proteins. Accordingly, 19 proteins qualified as 'membrane-associated', of which 14 could indeed be classified as 'membrane-bound', based on the stringency of the criteria met by them (Table 1). Three proteins of the latter category were lipoproteins (Rv0934, Rv1270c and Rv1368). Rv1270c and Rv1368 are members of the P4.24 family of lipoproteins, which show strong sequence conservation (**http://genolist.pasteur.fr/TubercuList/mast/P4.24).** This family also includes Rv1411c and Rv2945c. The third lipoprotein detected here, Rv0934, is the well-characterized 38 kDa antigen that is the 'membrane-bound' substrate binding component of the ABC transporter required for phosphate uptake (Braibant *et al*., 2000). We had previously identified this protein in the 'integral membrane protein' pools of BCG (Mehrotra *et al*., 1999) and *M. tuberculosis* (unpublished) with the help of monoclonal antibodies. Previous proteomic analyses of *M. tuberculosis* have not detected any lipoprotein, although one study (Rosenkrands *et al*., 2000b) has detected the 38 kDa (Rv0934) and 19 kDa lipoproteins through staining with antibodies.

We have classified some of the proteins as membrane-associated, mainly on the basis of published reports, even though their structural attributes do not suggest a membrane origin. These observations point to that fact that structure alone may not be the deciding factor as far as the association of proteins with the cell membrane is concerned. The proteins may be bound to the membrane simply to fulfil their functional obligations. Consequently, they could become part of multimeric complexes involving membrane proteins and may not dissociate easily under the conditions of sample preparation. For instance, this could be true of FadA2 (Rv0243), which is involved in lipid metabolism — a biochemical process occurring in membranes — and Rv1872c, which is a probable lactate dehydrogenase. This enzyme generally interacts with membrane-bound components of the electron transfer chain of bacteria. Heat-shock proteins are also known to become associated with membrane proteins while working as molecular chaperones (Cordwell *et al*., 2001; Wissing *et al*., 2000). This could be the reason behind the observed presence of four HSPs (Rv2031c, $\alpha$-crystallin; Rv0251c, HSP20; Rv3417c, groEL1; and Rv0440c, groEL2) in our membrane preparations. The $\alpha$-crystallin homologue has already been reported to be membrane-associated in our studies (Mehrotra *et al*., 1999) and those of others (Young and Garbe, 1991; Lee *et al*., 1992). Other identified proteins that could be associated with the membrane in this way are bacterioferritin (bfrA, Rv1876) and ATP synthase (Rv1308, Rv1309, Rv1310). Bacterioferritin is a conserved, iron-rich protein previously described as the major membrane protein-II (MMP-II) of *M. leprae* (Pessolani *et al*., 1994). Membrane association of ATP synthase is also well documented (Futai *et al*., 1989). Similar reasons could be used to explain the presence of some other, apparently cytosolic, proteins in the membrane. On the other hand, some truly cytosolic proteins may simply become entrapped within membrane vesicles formed during the sonication process and become difficult to remove by the extraction methods used (Friso and Wikstrom, 1999).

In view of the reports recommending one method or the other, it was important to compare the efficacies of the detergent (Triton X114) and carbonate extraction procedures in uncovering putative membrane proteins of the bacillus. At the outset, there was hardly any resemblance between the profiles revealed by the two procedures, in terms of either 2D patterns or identified proteins. Only two proteins, both HSPs (Rv2031c and Rv0251c), were commonly present in the two preparations. The results suggest a better efficacy of the detergent-based method in selecting membrane proteins. 60% of all identified proteins of the detergent extract qualified as 'membrane bound' (Table 1) whereas only 26% of proteins from the carbonate extracted sample did so. More importantly, the detergent-soluble but not carbonate-extracted sample displayed three lipoproteins of the bacillus, as elaborated above. These observations appear logical, since hydrophobic/lipophilic proteins, such as those from the membrane, are expected to partition selectively in a detergent-rich medium. The success of the carbonate-based strategy reported

Copyright © 2002 John Wiley & Sons, Ltd. 

in some cases (Molloy *et al.*, 2000; Fujiki *et al.*, 1982) could depend on the complexity of the target membrane. The mycobacterial cell envelope (including the membrane) is considered unique in its composition and complexity (Barksdale and Kim, 1977; Brennan and Nikaido, 1995). Even within the genus, the envelope of 'slow growers', such as *M. tuberculosis*, is more complex that that of the 'fast growers'. Many a 'sticky' and poorly defined molecule, such as lipoarabinomannans, arabinogalactans, phosphatidyl inositol mannosides, etc., and several others (mostly undefined), such as proteoglycolipids, glycolipids, phospholipids, sulpholipids and glycans, are present in the cell wall or membrane. These components, besides imposing problems on membrane solubilization, have most probably also contributed to the streaking and smearing seen in our 2D gels. Even in one-dimensional (1D) gels, where a strong detergent like SDS is used for sample solubilization, the smearing effect is observed (Lee *et al.*, 1992; Mehrotra *et al.*, 1999).

The reliability of the protocol for comparative analyses of mycobacterial membrane proteins was also an important consideration in this study. 2D protein profiles of both *M. tuberculosis* and BCG membranes appeared to be similar, reflecting the genomic similarities between these microbes (Gordon *et al.*, 1999). The identity of a low molecular mass protein, which was expressed prominently in *M. tuberculosis* but not at all in BCG, was revealed as ESAT-6 by peptide mass mapping. The genome of *M. tuberculosis* H37Rv has five copies of a cluster of genes known as the ESAT-6 (early secretory antigenic target) loci. These clusters contain members of the ESAT-6 gene family (encoding secreted T cell antigens that lack detectable secretion signals), as well as genes encoding secreted, cell-wall-associated serine proteases, putative ABC transporters, ATP-binding proteins and other membrane-associated proteins (Tekaia *et al.*, 1999). The multiple duplicates of the ESAT-6 gene of *M. tuberculosis* are also conserved in other mycobacteria, e.g. *M. bovis*, *M. leprae*, *M. avium* and the avirulent strain *M. smegmatis*. The protein in *M. leprae* appears in the cell wall fraction (Spencer *et al.*, 2002). Thus there is 'circumstantial' evidence for the existence of this protein in the cell membrane. ESAT-6 is not expected to be present in BCG, since the corresponding gene locus (RD1) of *M. tuberculosis* is deleted in BCG (Gordon *et al.*, 1999).

In conclusion, the knowledge of the proteome of *M. tuberculosis* is still in a descriptive phase and there is a long way to go before the complete definition of the proteins allows identification of candidate drug targets, diagnostic probes or components of a vaccine. Comparative proteomics of the highly conserved *M. tuberculosis* complex is a powerful approach to understand phenotypic differences such as virulence (Betts *et al.*, 2000; Mattow *et al.*, 2001b) and merits further application. The area of membrane proteomics is only just beginning to unfold. Clearly, progress in this area hinges not only on our ability to efficiently solubilize the membrane proteins but also on making them free from interfering substances (Santoni *et al.*, 2000). Nonetheless, the approach using Triton X114-based extraction has shown promise in this as well as earlier (Wissing *et al.*, 2000) studies. We have provided evidence for some of the 'true' membrane proteins of the bacillus and also detected some of those described as 'unknowns' or 'hypotheticals' in the genome database.

### Acknowledgements

This work was supported by grants from the Indo-French Centre for Promotion of Advanced Research (IFC-PAR 2303-2), the European Union (QLRT-2000-02018), the Institut Pasteur and the Association Française Raoul Follereau. We thank Dr Thierry Rabilloud (CEA-Grenoble, France) for helpful discussions. S.A. and K.K. are thankful to CSIR, India, for their Senior and Junior Research Fellowships, respectively. This paper is CDRI Communication No. 6177.

### References

Akins DR, Purcell BK, Mitra MM, *et al.* 1993. Lipid modification of the 17 kDa membrane immunogen of *Treponema pallidum* determines macrophage activation as well as amphiphilicity. *Infect Immun* **61**: 1202–1210.

Barksdale L, Kim K-S. 1977. Mycobacterium. *Bacteriol Rev* **41**: 217–372.

Betts JC, Dodson P, Quan S, *et al.* 2000. Comparison of the proteome of *Mycobacterium tuberculosis* strain H37Rv with clinical isolate CDC 1551. *Microbiology* **146**: 3205–3216.

Bordier C. 1981. Phase separation of integral membrane proteins in Triton X-114 solution. *J Biol Chem* **256**: 1604–1607.

Braibant M, Gilot P, Content J. 2000. The ATP binding cassette (ABC) transport systems of *Mycobacterium tuberculosis*. *FEMS Microbiol Rev* **24**: 449–467.

Brennan PJ, Nikaido H. 1995. The envelope of mycobacteria. *Ann Rev Biochem* **64**: 29–64.

Copyright © 2002 John Wiley & Sons, Ltd. 

Cole ST, Brosch R, Parkhill J, *et al*. 1998. Deciphering the biology of *Mycobacterium tuberculosis* from the complete genome sequence. *Nature* **393**: 537–544.

Cordwell SJ, Nouwens AS, Walsh BJ. 2001. Comparative proteomics of bacterial pathogens. *Proteomics* **1**: 461–472.

Covert BA, Spencer JS, Orme IM, *et al*. 2001. The application of proteomics in defining the T cell antigens of *Mycobacterium tuberculosis*. *Proteomics* **1**: 574–586.

Deres KH, Schild H, Weismuller K-H, *et al*. 1989. *In vivo* priming of virus-specific cytotoxic T lymphocytes with synthetic lipopeptide vaccine. *Nature* **342**: 561–564.

Domenech P, Barry CE III, Cole ST. 2001. *Mycobacterium tuberculosis* in the post-genomic age. *Curr Opin Microbiol* **4**: 28–34.

Frankenburg F, Axelrod O, Kutner S, *et al*. 1996. Effective immunization of mice against cutaneous leishmaniasis using an intrinsically adjuvanted synthetic lipopeptide vaccine. *Vaccine* **14**: 923–929.

Friso G, Wikstrom L. 1999. Analysis of proteins from membrane-enriched cerebellar preparations by two-dimensional gel electrophoresis and mass spectrometry. *Electrophoresis* **20**: 917–927.

Fujiki Y, Hubbard AL, Fowler S, *et al*. 1982. Isolation of intracellular membranes by means of sodium carbonate treatment: application to endoplasmic reticulum. *J Cell Biol* **93**: 97–102.

Futai M, Noumi T, Maeda M. 1989. ATP synthase ($H^+$ -ATPase): results by combined biochemical and molecular biological approaches. *Ann Rev Biochem* **58**: 111–136.

Gordon SV, Brosch R, Billault A, *et al*. 1999. Identification of variable regions in the genomes of tubercle bacilli using bacterial artificial chromosome arrays. *Mol Microbiol* **3**: 643–655.

Gorg A, Obermaier C, Boguth G, *et al*. 2000. The current state of two-dimensional electrophoresis with immobilized pH gradients. *Electrophoresis* **21**: 1037–1053.

Jensen ON. 2000. Modification-specific proteomics: systematic strategies for analysing post-translationally modified proteins. In *Proteomics: A Trends Guide*. Elsevier: Amsterdam; 36–42.

Jungblut PR, Muller EC, Mattow J, *et al*. 2001. Proteomics reveals open reading frames in *Mycobacterium tuberculosis* H37Rv not predicted by genomics. *Infect Immunol* **69**: 5905–5907.

Jungblut PR, Schaible UE, Mollenkopf HJ, *et al*. 1999. Comparative proteome analysis of *Mycobacterium tuberculosis* and *Mycobacterium bovis* BCG strains: towards functional genomics of microbial pathogens. *Mol Microbiol* **33**: 1103–1117.

Kyte J, Doolittle RF. 1982. A simple method for displaying the hydropathic character of a protein. *J Mol Biol* **157**: 105–132.

Laemmli UK. 1970. Cleavage of structural proteins during the assembly of the head of bacteriophage T4. *Nature* **227**: 680–685.

Lee B, Hefta SA, Brennan PJ. 1992. Characterization of the major membrane protein of virulent *Mycobacterium tuberculosis*. *Infect Immun* **60**: 2066–2074.

Markwell MAK, Haas SM, *et al*. 1978. A modification of the Lowry procedure to simplify protein determination in membrane and lipoprotein samples. *Anal Biochem* **87**: 206–210.

Mattow J, Jungblut PR, Müller EV, *et al*. 2001a. Identification of acidic, low molecular mass proteins of *Mycobacterium tuberculosis* strain H37Rv by matrix-assisted laser desorption/ionization and electrospray ionization mass spectrometry. *Proteomics* **1**: 494–507.

Mattow J, Jungblut PR, Schaible UE, *et al*. 2001b. Identification of proteins from *Mycobacterium tuberculosis* missing in attenuated *Mycobacterium bovis* BCG strains. *Electrophoresis* **22**: 2936–2946.

Max Planck Institute Protein Database: **http://www.mpiib-berlin.mpg.de/2D-PAGE**

Mehrotra J, Bisht D, Tiwari VD, *et al*. 1995. Serological distinction of integral plasma membrane proteins as a class of mycobacterial antigens and their relevance for human T cell activation. *Clin Exp Immunol* **102**: 626–634.

Mehrotra J, Mittal A, Dhindsa MS, *et al*. 1997. Fractionation of mycobacterial integral membrane proteins by continuous elution SDS–PAGE reveals the immunodominance of low molecular weight subunits for human T cells. *Clin Exp Immunol* **109**: 446–450.

Mehrotra J, Mittal A, Rastogi AK, *et al*. 1999. Antigenic definition of plasma membrane proteins of Bacillus Calmette–Guerin: predominant activation of human T cells by low-molecular-mass integral proteins. *Scand J Immunol* **50**: 411–419.

Molloy MP, Herbert BR, Slade MB, *et al*. 2000. Proteomic analysis of the *Escherichia coli* outer membrane. *Eur J Biochem* **267**: 2871–2881.

Molloy MP, Herbert BR, Walsh BJ, *et al*. 1998. Improved protein solubility in two-dimensional electrophoresis using tributyl phosphine as reducing agent. *Electrophoresis* **19**: 837–844.

Monahan I, Betts J, Banerjee D, *et al*. 2001. Differential expression of mycobacterial proteins following phagocytosis by macrophages. *Microbiology* **147**: 459–471.

Pessolani MC, Smith DR, Rivoire B, *et al*. 1994. Purification, characterization, gene sequence, and significance of a bacterioferritin from *Mycobacterium leprae*. *J Exp Med* **180**: 319–327.

ProtParam tool: **http://us.expasy.org/tools/protparam.html**

PSORT: **http://psort.nibb.ac.jp**

Raviglione MC, Snider DE, Kochi A. 1995. Global epidemiology of tuberculosis. morbidity and mortality of a worldwide epidemic. *J Am Med Assoc* **273**: 220–226.

Rosenkrands I, King A, Weldingh K, *et al*. 2000a. Towards the proteome of *Mycobacterium tuberculosis*. *Electrophoresis* **21**: 3740–3756.

Rosenkrands I, Weldingh K, Jacobsen S, *et al*. 2000b. Mapping and identification of *Mycobacterium tuberculosis* proteins by two-dimensional gel electrophoresis, microsequencing and immunodetection. *Electrophoresis* **21**: 935–948.

Santoni V, Molloy M, Rabilloud T. 2000. Membrane proteins and proteomics: Un amour impossible? *Electrophoresis* **21**: 1054–1070.

Shevchenko A, Wilm M, Vorm O, *et al*. 1996. Mass spectrometric sequencing of proteins silver-stained polyacrylamide gels. *Anal Chem* **68**: 850–858.

Sigler K, Hofer M. 1997. Biotechnological aspects of membrane function. *Crit Rev Biotechnol* **17**: 69–86.

Snider DE, Castro KG. 1998. The global threat of drug-resistant tuberculosis. *New Engl J Med* **338**: 1689–1690.

Spencer JS, Marques MA, Lima MC, *et al*. 2002. Antigenic specificity of the *Mycobacterium leprae* homologue of ESAT-6. *Infect Immun* **70**: 1010–1013.

Statens Serum Institut Protein Database: **http://www.ssi.dk/publichealth/tbimmun**

Copyright © 2002 John Wiley & Sons, Ltd. 

Tekaia F, Gordon SV, Garnier T, *et al*. 1999. Analysis of the proteome of *Mycobacterium tuberculosis in silico*. *Tubercle Lung Dis* **79**: 329–342.

TMPRED: **http://www.ch.embnet.org/software/TMPRED_form.html**

Tuberculist: **http://genolist.pasteur.fr/TubercuList**

Wissing J, Heim S, Flohe L, *et al*. 2000. Enrichment of hydrophobic proteins via Triton X-114 phase partitioning and hydroxyapatite column chromatography for mass spectrometry. *Electrophoresis* **21**: 2589–2593.

Young DB, Duncan K. 1995. Prospects for new interventions in the treatment and prevention of mycobacterial disease. *Ann Rev Microbiol* **49**: 641–673.

Young DB, Garbe TR. 1991. Lipoprotein antigens of *Mycobacterium tuberculosis*. *Res Microbiol* **142**: 55–65.

Copyright © 2002 John Wiley & Sons, Ltd. 
